# Supplementary material for: Quantum electrometer for time-resolved material science at the atomic lattice scale
Source: Nat Commun. 2025 Jul 11;16:6435. doi: 10.1038/s41467-025-61839-2 (PMC12254403; doi:10.1038/s41467-025-61839-2)
Supplement: Supplementary file 1 — Supplementary Information [file 41467_2025_61839_MOESM1_ESM.pdf]

Supplementary Information to Quantum Electrometer for Time-Resolved Material Science at the Atomic Lattice Scale

Gregor Pieplow,[1,*] Cem Güney Torun,[1,*] Charlotta Gurr,[1] Joseph H. D. Munns,[1] Franziska Marie Herrmann,[1] Andreas Thies,[2] Tommaso Pregnolato,[1,2] and Tim Schröder[1,2,†]

[1]*Department of Physics, Humboldt-Universität zu Berlin, 12489 Berlin, Germany*
[2]*Ferdinand-Braun-Institut (FBH), Gustav-Kirchhoff-Str. 4, 12489 Berlin, Germany*

SIMULATION DETAILS

Relative electric field sensitivity

The relative electric field sensitivity $\delta\epsilon = \Delta E/E_\mathrm{s}$ at a given ρ_trap can be calculated by identifying ΔE corresponding to the smallest spectral shift Δ_Stark that can be resolved according to a modified Rayleigh criterion[1] as described below.

We calculate ΔE in a two step procedure: First we simulate the expected inhomogeneously broadened linewidth in the presence of an electric field $\mathbf{E}_\mathrm{s}$ (see Supplementary Fig. 1), which is generated by either a charged proximity trap or by a non-neutral charge state of the entire spatial trap configuration. The total field at the sensor position can be separated into two components $\mathbf{E} = \mathbf{E}_\mathrm{s} + \delta\mathbf{E}_\mathrm{s}$, where $\delta\mathbf{E}_\mathrm{s}$ is a fluctuating electric field produced by the varying charge states of the remote trap configuration. Similar modeling has been performed in 2.

The average value of the non-linear Stark shift is given by $\langle\Delta_\mathrm{Stark}\rangle = -\Delta\alpha(E_\mathrm{s}^2+\sigma^2)$. Its variance is $\sigma_{\Delta_\mathrm{Stark}} = \Delta\alpha^2(4E_\mathrm{s}^2\sigma^2 + 2\sigma^4)$ (assuming $\delta\mathbf{E}_\mathrm{s}$ is normally distributed with variance σ). The expressions demonstrate that a field induces both a discrete spectral shift and a quasi-permanent dipole moment, resulting in inhomogeneous broadening of lines dependent on the magnitudes of E_s and σ.

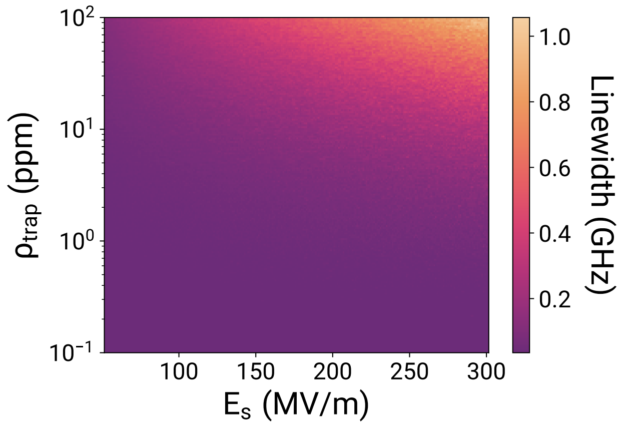

Supplementary Figure 1. **Inhomogeneous broadening as a function of the local bias field** E_s. The linewidth increases due to an increasing E_s or ρ_trap.

In the second step, we calculate ΔE at a given ρ_trap by using a modified Rayleigh criterion: Two spectral peaks originating from distinct fields E_s and E'_s are considered separable if the sum of the two individually normalized lineshapes resulting from $\mathbf{E} = \mathbf{E}_\mathrm{s} + \delta\mathbf{E}_\mathrm{s}$ and $\mathbf{E} = \mathbf{E}'_\mathrm{s} + \delta\mathbf{E}_\mathrm{s}$ exhibit a contrast of at least 26.3% between their local maxima.

For Fig. 2c from the main text, we choose $\mathbf{E}_\mathrm{s} = (0, 0, E_\mathrm{s})$. To determine the $\langle\Delta_\mathrm{Stark}\rangle$ and the inhomogeneously broadened linewidth we employ a Monte Carlo simulation as outlined in the simulation overview. The traps generating $\delta\mathbf{E}_\mathrm{s}$ were placed at a fixed density ρ_trap in a conical volume $z > 0$ with an opening angle of 45°, mimicking the anisotropic distribution of traps produced by implantation and annealing (e.g. Fig. 5 from the main text). The conical volume was capped at $z = 30$ nm. A spherical volume with a radius of 2.5 nm, was left empty of traps to reduce the occurrence of exaggerated multimodal spectral features.

The averaged linewidths required for Fig. 1c from the main text are calculated using $\gamma_\mathrm{FWHM} = a\sigma_\mathrm{hom} + (b\sigma_\mathrm{hom}^2 + \sigma_\mathrm{inhom}^2)^{1/2}$, where σ_hom and σ_inhom are the full width half maximum of the Lorentzian and Gaussian Contribution to the Voigt profile and $a = 0.5346, b = 0.2166$[3]. In total we average the Gaussian and Lorentzian components of 100 different spatial trap configurations at a given ρ_trap. For each ρ_trap we sample 2500 randomly generated charge states to simulate a single spectrum. The averaged spectral profiles corresponding to $\mathbf{E} = \mathbf{E}_\mathrm{s} + \delta\mathbf{E}_\mathrm{s}$ and $\mathbf{E}' = \mathbf{E}'_\mathrm{s} + \delta\mathbf{E}_\mathrm{s}$ are then used to determine $\Delta\mathbf{E}_\mathrm{s} = |E_\mathrm{s} - E'_\mathrm{s}|$ using the Rayleigh criterion.

Finally, we calculate the relative sensitivity shown in Fig. 2c from the main text by dividing $\delta\epsilon = \Delta E/E_\mathrm{s}$ at a given ρ_trap.

Impact of noise

The relative electric field sensitivity shown in Fig. 1c from the main text depends on how well the center frequency of a spectral peak can be determined. The determination of the peak position is affected by uncertainties induced by noise other than the stochastic shifts of the C-transition. Sources for such noise can be dark counts of the detector or undesired background fluorescence. In this section we estimate signal to noise ratios (SNR) that are required to enable the relative sensitivities shown in Fig. 1c (main text). We first assume that the α dominates the response of the sensor's interaction with

* These authors contributed equally to this work
† Corresponding author: tim.schroeder@physik.hu-berlin.de

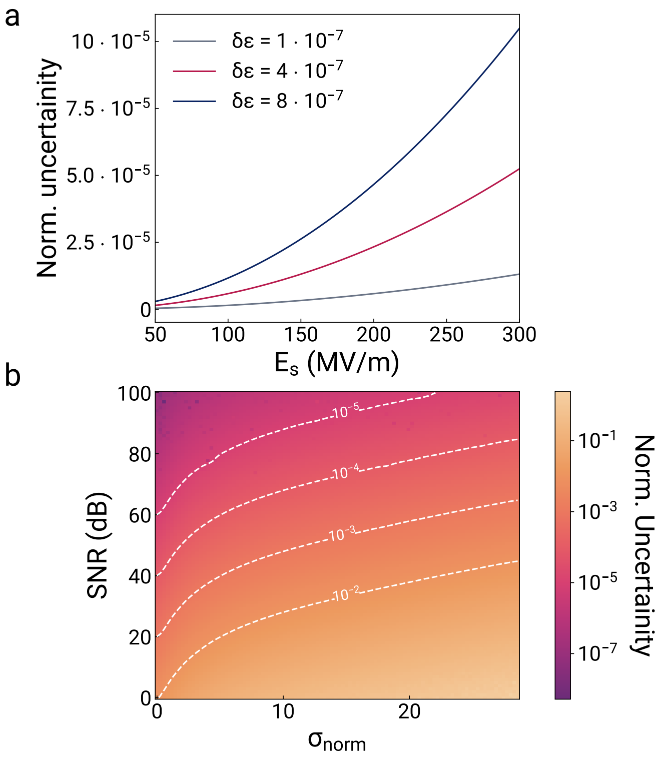

Supplementary Figure 2. **Normalized uncertainty.** **a** The uncertainty normalized to the homogeneous linewidth for three different values of the relative electric field sensitivity $\delta\epsilon = 1, 4, 7 \cdot 10^{-7}$. **b** The uncertainty extracted from a fit as a function of the SNR and the Gaussian component of the Voigt profile, both normalized to the homogeneous linewidth.

an electric field, so that the relative Stark shift (Eq. (1), main text) produced by two distinct resolvable electric fields E_1 and E_2 becomes

$$|\delta\omega| \approx \frac{\alpha}{2}|E_1^2 - E_2^2| \quad (1)$$

$$= \alpha\delta\epsilon E_1^2 \,, \quad (2)$$

where we used the definition of the relative electric field sensitivity $\delta\epsilon = |E_1 - E_2|/E_1$ and assumed that $E_1 + E_2 \approx 2E_1$. We define the normalized uncertainty as $\Lambda = |\delta\omega|/\gamma_{\mathrm{hom}}$, where we chose the homogeneous linewidth of the SnV $\gamma_{\mathrm{hom}} = 35$ MHz as a reference. Λ is the smallest Stark shift difference that has to be resolved, so that a relative electric field sensitivity of $\delta\epsilon$ can be reached. In Supplementary Fig. 2a we show the normalized uncertainty for three values of $\delta\epsilon$, which are representative values picked from Fig. 1c from the main text. For the range of relevant field strengths, we find that $2.5 \cdot 10^{-5} < \Lambda \leq 10^{-4}$. To make sense of the normalized uncertainty we simulate the normalized uncertainty of the central peak position $\delta\omega_0/\gamma_{\mathrm{hom}}$ of a spectral fit with a centered Voigt profile $V(\omega - \omega_0, \gamma_{\mathrm{hom}}, \sigma)$ with $\omega_0 = 0$, a Lorentzian component γ_{hom} and Gaussian component σ in the presence of noise. If $\delta\omega_0/\gamma_{\mathrm{hom}}$ produced by the fit does not exceed the threshold demanded by Λ we assume the corresponding relative electric field sensitivity to be achievable. In Supplementary Fig. 2b we show the result of the simulations. We normalize the Gaussian component of the Voigt profile according to $\sigma_{\mathrm{norm}} = \sigma/\gamma_{\mathrm{hom}}$. We calculate $\mathrm{SNR} = 10\log_{10}(A^2/\delta^2_{\mathrm{noise}})$, where the amplitude of the Voigt profile $A = 1$ and $\delta^2_{\mathrm{noise}}$ is the amplitude of the white noise: $S(\omega) = V(\omega, \gamma_{\mathrm{hom}}, \sigma) + \delta_{\mathrm{noise}}$. Supplementary Fig. 2b shows the required SNR as a function of σ_{norm}. Even though the requirements are challenging, they are not a fundamental limitation of our proposed sensor. For the multimodal spectrum in Fig. 2 from the main text, the normalized uncertainties are between $10^{-2} \leq \Lambda < 7 \cdot 10^{-1}$. The poor Λ in our experiment is mostly due to experimental imperfections, and not a fundamental constraint of the sensor principle.

Even though the Λ in our implementation does not reach the simulated requirement to produce the simulated limit of the relative sensitivity of our proposed electrometer, they are sufficient for the claimed Angstrom resolution of the sensor. We can perform a similar estimation of the normalized sensitivity as a function of the relative resolution $\delta\epsilon_r = (r_1 - r_2)/r_1$ where we find making similar assumptions ($r_1 + r_2 \approx 2r_1$) as in the paragraph above so that

$$\Lambda = 2\delta\epsilon_r a^2 \frac{\alpha}{\gamma}\left(\frac{1}{r_{\mathrm{bias}}^2 r_1^2} + \frac{1}{r_1^4}\right) , \quad (3)$$

where $a = 1/4\pi\epsilon_0\epsilon_r$. We find that $18 < \Lambda < 166$, for $\delta\epsilon_r = 1$, $r_{\mathrm{bias}} = 10$ Å and $r_1 \in (10, 30)$ Å, which far exceeds the relative fit uncertainties provided in the paragraph above.

Resolution

For determining the spatial resolution $\Delta_{\mathrm{r}} = |\mathbf{r} - \mathbf{r}'|$, where $\mathbf{r}$ and $\mathbf{r}'$ are two distinct positions of point-like charges, we perform the same calculation as for the relative electric field sensitivity but additionally assume that the charges generate electric fields

$$\mathbf{E}(q, \mathbf{r}) = \frac{q_i}{4\pi\epsilon_0\epsilon_r}\frac{\mathbf{r}}{r^3} , \quad (4)$$

where ϵ_0 is the vacuum permittivity and $\epsilon_r = 5.5$ is the relative permittivity of diamond. Using the bulk expression and neglecting surface contributions is justified because of the pillar dimensions $r > 40$ nm (Fig. 4b,c from the main text), if the SnV is located on the pillar's symmetry axis.

In Fig. 1d from the main text, we present the sensor's resolution in the presence of a constant static field $\mathbf{E}_{r_0}$ produced by a negatively charged trap situated at the fixed location $\mathbf{r}_0 = (0, 0, 0.8)$ nm.

As described in the previous section, Fig. 1d (main text) was generated in a two step procedure: First the expected spectral profiles were computed for a given ρ_{trap} and $\mathbf{E} = \mathbf{E}(-1, \mathbf{r}_0) + \mathbf{E}(-1.\mathbf{r}_1) + \delta\mathbf{E}_{\mathrm{s}}$. Then the resolution was calculated by employing the Rayleigh criterion.

We place $\mathbf{r}_1 = (0, 0, d)$ in line with $\mathbf{r}_0$. The averaged profiles are then used to determine the smallest resolvable distance $\Delta_{\mathrm{r}} = |\mathbf{r} - \mathbf{r}'|$ from the spectral profiles corresponding

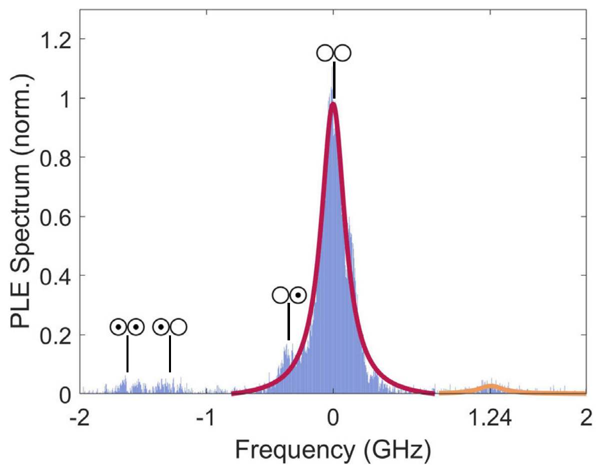

Supplementary Figure 3. **Experimental estimation of the bias field on our sensor.** Background subtracted integrated spectrum of the linescans between (0-200) from the Fig. 2b from the main text. The fit centered at 1.24 GHz indicates the existence of an extra charge trap which is ionized most of the time.

to the fields $\mathbf{E}_{\text{bias}} = \mathbf{E}(-1, \mathbf{r}_0) + \mathbf{E}(-1, \mathbf{r}_1) + \delta\mathbf{E}_{\text{s}}$ and $\mathbf{E}_{\text{bias}} = \mathbf{E}(-1, \mathbf{r}_0) + \mathbf{E}(-1, \mathbf{r}'_1) + \delta\mathbf{E}_{\text{s}}$.

Most likely spatial trap configuration

The integrated multimodal spectrum in Fig. 2b from the main text can arise from distinct spatial charge configurations, leading to identical results. Nonetheless, it is possible to narrow down potential proximity charge configurations.

The integrated spectrum of Fig. 2b (main text) shows four peaks. The two simplest configurations producing such a spectrum are: A) three traps, where one trap is permanently charged and the other two can be in the states $[\bigcirc\bigcirc, \bigcirc\odot, \odot\bigcirc, \odot\odot]$ or B) four proximity traps with one trap being permanently charged and the other three in the charge states $[\bigcirc\bigcirc\bigcirc, \bigcirc\bigcirc\odot, \bigcirc\odot\bigcirc, \odot\bigcirc\bigcirc]$. In both cases, a bias field / permanently charged trap is required to explain the inhomogeneous broadening of the rightmost peak. Many more trap configurations could in principle produce the same features, but we deem them less likely, given that they require more and more traps, where only a subset of all possible charge state combinations then contributes to the observed spectrum. Of the two scenarios, scenario A) requires the fewest additional assumptions.

The strongest argument in favor of A) is based on the transition probability $p(\bigcirc\bigcirc \rightarrow \odot\odot) = 3(1)\%$ (see Supplementary Fig. 7). If one assumes that traps independently ionize with a probability P, then the corresponding rates for B) $p(\bigcirc\bigcirc\bigcirc \rightarrow \odot\bigcirc\bigcirc) \approx P$. However, it is one of the least likely processes. Scenario A) would require two ionization events which would be of order P^2, which is much closer to the observation. The same argument can be constructed for $p(\bigcirc\odot \rightarrow \odot\odot) = 33(6)\%$. For B) the corresponding event would be $p(\bigcirc\odot\bigcirc \rightarrow \odot\bigcirc\bigcirc) \approx P^2$, which should be unlikely. However, the single ionization event $p(\bigcirc\odot \rightarrow \odot\odot)$ is more likely and therefore more consistent with the two trap scenario.

We estimate the bias field experienced by the sensor by positioning a constantly ionized charge trap such that the inhomogeneous broadening of the simulations matches the observed linewidths. From the simulations, we find a bias field inducing a spectral shift of 1.3(4) GHz. We compare this result with the integrated spectrum for lines between 0 and 200 from the spectrum from Fig. 2b (main text) and find evidence of a small blue shifted peak with a spectral shift of 1.24(2) GHz (Supplementary Fig. 3) compared to the $\bigcirc\bigcirc$ peak. This experimentally substantiates that there is indeed a third charge trap which is ionized most of the time. Analyzing the inhomogenous linewidth with Monte Carlo simulations and experimental data independently affirm each other on the estimated magnitude of the bias field. This consistency further demonstrates that our simulations can replicate the charge environment and is able to detect traps that do not dynamically change their charge on the time scales of the remote traps.

Generalized algorithm for trap configuration determination

Here, we provide a generalized algorithm for determining a charge trap *configuration* using the jumping probabilities between different spectral peaks, i.e. trap *states*.

Definitions: Trap state: A possible distinguishable combination of ionized traps.

Trap configuration: The total set of trap states the sensor detects.

Correlation matrix: The probability matrix of trap states changing from one to another, i.e., a spectral jump occurring from one peak to another. It has a total of M*(M-1) entries where we discard the diagonal (no state change) elements. We order the peaks (trap states) from the least red-shifted (no ionization) to most red-shifted.

Algorithm: i. Data acquisition: Automatically perform a series of photoluminescence excitation (PLE) scans of the probe.

ii. Spectral analysis: Utilize a peak-finding algorithm to identify peaks within the integrated spectra for which each peak corresponds to a trap state. It is possible to return to step i, and apply RORO sequences for enhancing the temporal resolution.

iii. Correlation matrix extraction: Extract jumping probabilities from the individual and time-resolved PLE scans. The jumping probabilities are only associated to the peaks that were identified in step ii. This is explained in detail for the temporal analysis of the S1 data.

iv. Determining the trap configuration:

The number of peaks in the spectrum = M

The number of detectable traps that can host a charge in the vicinity of the sensor = N

We assume single-ionization events exist and are more likely than multi-ionization events. We ensure the scan speed is high enough such that a maximum of two peaks

are observed in between scans. Based on this, we assume that a single ionization process could have happened in this time period. We attribute anything below a threshold (e.g. 3%/M) as a multi-ionization event occurring in a single scan and neglect it (set it to 0). This reduces the complexity of detectable trap configurations. From the M, we determine the minimal number of N with M> 2^{N-1} and the maximal number, N=M. To determine N, we calculate the order (number) of correlated (nonzero probability) steps of the jumping events. We analyze the jumping probabilities from the non-ionized case '0' (least red-shifted peak) and step by step reach a particular peak 'k': p($0 \rightarrow 1 \rightarrow \ldots k-3$ steps$\ldots \rightarrow k$).

Example application for M peaks:

• We expect the simplest configuration of traps to consist of N=M-1 traps with N trap states, i.e., no multi-ionized traps at the same time visible in the spectrum, and only single ionization events are likely. This corresponds to 2*(M-1) nonzero values in the correlation matrix that are all correlated to the least red-shifted peak.

• Under similar assumptions, we can construct a spectrum from N=M-2 traps where M-2 peaks are the results of ionizing M-2 single traps. One peak corresponds to no ionization. The remaining peak has to emerge from one trap state with two ionized traps. This can already be distinguished from the N=M-1 scenario, by identifying 4 entries in the correlation matrix that are bigger than 0 that are not correlated with the least red-shifted peak (first column, first row). These nonzero elements uniquely correlate each peak to a trap state.

• With the N=M-3 traps case, we have M-3 peaks correlated with the single ionized traps states. One peak corresponds to non-ionized trap state. The remaining two peaks can be associated to two configurations with either two distinguishable simultaneously ionized traps states or two trap states which include two and three simultaneously ionized trap states. These two possible trap configurations from each other are further distinguished by the higher order correlations revealing whether or not subsequent ionization processes have occurred. By considering these higher-order correlations, it is in principle possible to further identify which set of trap states is responsible for the observed peaks.

• This method can be generalized to further reduce the number of participating traps, by identifying the highest-order correlation. By comparing the observed correlations with the correlation matrices we can identify a trap configuration with the experimentally observed data.

v. Determining remote charge trap distribution: The remote trap distribution (density, spatial distribution) can be predefined or left variable and fine-tuned through a series of preliminary Monte Carlo scans to model the integrated spectrum, without requiring manual input. In this paper, the spatial distribution of the remote trap density was estimated with a physical model of the implantation damage distribution. Other physical models can be also supplemented to the simulations. Without a physical model, a predefined charge distribution (such as cylindrically symmetric) can also be used.

vi. Estimating the proximity charge trap positions: Based on the selected trap configuration there are two possible ways of determining the proximity charge trap positions. First comes the initialization of the trap positions at random positions with the distances corresponding to the spectral shifts of the assigned peaks. These distances are determined by a set of equations that govern the DC Stark shifts. The remaining free parameters (relative angles and distances) are further refined by minimizing a Chi-squared test as explained in detail in the corresponding sections. Based on this test, the most probable trap configuration and positions can be returned in a general and automated fashion.

Annealing

The creation of V_2 is understood to be a consequence of implantation damage[4,5] and the annealing procedure: Implantation damage occurs during the collision cascade in the diamond lattice that decelerates the implanted ion. Collisions with an energy above the displacement-threshold ($\approx$ 37.5 − 47.6 eV[6] much smaller than typical implantation energies) dislodge carbon atoms and produce Frenkel pairs: a pair of V_1 and a dislocated carbon atom located at an interstitial lattice site. After the implantation, an annealing procedure is performed to create the color center through vacancy diffusion and to heal the lattice damage. At temperatures above 600 K interstitial carbon becomes mobile[7] and at 800 K the V_1[4] have a high degree of mobility. Consequently, during annealing, interstitial carbon can either recombine with the V_1[8,9] or diffuse away from the damage site and eventually leave the sample through the boundaries. The V_1 that have not recombined with an interstitial carbon can form immobile V_2[4], vacancy clusters, or create a color center together with the implanted ion.

Our model assumes one mobile species (V_1) and considers the formation of V_2 without multi-vacancy complexes. Since we do not consider multiple species, we forego assigning different hopping frequencies as in[10]. The initial number N and the 3D distribution of the V_1 after implantation is estimated using a SRIM simulation[11]. Assuming a percentage of V_1 not being consumed by interstitial carbon, which we call yield quantified in % of Frenkel pairs that have not recombined (V_1 yield in Fig. 5 from the main text) we find a range of concentrations of V_2 shown in Fig. 5 (main text) for the three atomic G4V species Si, Ge and Sn and varying implantation energies. We estimate the distribution of V_2 in the sample by using a kinetic Monte Carlo simulation. In each time step of the kinetic simulation, the V_1 can take a random step along any of the neighboring lattice sites. If two V_1 are adjacent to each other, they form a static V_2 which no longer diffuses. The initial distribution of V_1 is estimated using SRIM[11]. For each implantation energy we use 50000 implantation events for a given atomic species and implantation energy to find the probability distribution $p(z)$ of V_1 as a function of the depth z measured relative to the diamond surface (001). We then use the $p(z)$ to generate a realization of a spatial distribution of V_1 after a single implantation event. The V_1 are distributed on the diamond lattice according to the $p(z)$ along a narrow damage channel

with a rectangular cross section of $2a \times 2a$ as described in the main text. The loss of V_1, that do not contribute to the formation of V_2 due to recombination with interstitial carbon atoms is by reducing the initial amount of V_1 as determined by the SRIM simulation by a fixed percentage.

Bulk charges

Based on our model, we provide an overview of the charge trap densities and the resulting inhomogeneous broadening with certain thresholds enabling 90% interference visibility and > 87% entanglement fidelity according to 12. First, we use our Monte Carlo simulation to determine the distributions of linewidths for a given trap density ρ. We assume a carbon density of $\rho_C = 8/a^3$ in bulk and an isotropic distribution of traps in the environment of the SnV at a given density ρ. For each ρ we consider 500 spatial trap configurations that produce single peaked spectra for $\rho \in (1, 100)$ ppm. We use Supplementary Eq. (4) in the sum Eq. (11) (main text, methods) to calculate the spectra.

Surface charges

The surface density for both the semi-infinite half space and the cylindrical geometry given in ppm is calculated with respect to a carbon density of $\rho_c = 2/a^2$ [(001) plane]. For the semi infinite half space the traps are randomly positioned on a square with a 100 nm edge length. The cylindrical surface has a height of 100 nm. The simulation of the inhomogeneous linewidth was performed in both cases with the previously explained Monte Carlo method using 5000 different charge configurations for a single spatial configuration of traps. We also use the electrostatic fields of a point charge on a surface taking the respective boundary conditions into account. The electric field of a charge located on the surface of the semi infinite half space is

$$\mathbf{E}(q, \mathbf{r}_q) = \frac{q}{2\pi\epsilon_0(\epsilon_r + 1)} \frac{\mathbf{r}_q}{r_q^3} \tag{5}$$

For the cylindrical surface, we assume a diamond cylinder with radius R extending to $z = \pm\infty$. We used the expression in 13 [suppl. in 14] for the electric field of a charge on the cylindrical diamond surface. Here, we do not consider band bending, which can be advantageous for eliminating surface noise through screening. We also neglect free carrier screening, because we do not see the stark reduction of sensitivity to charge-noise that would be expected even for moderate screening lengths of tens of nanometers.

EXPERIMENTAL DETAILS

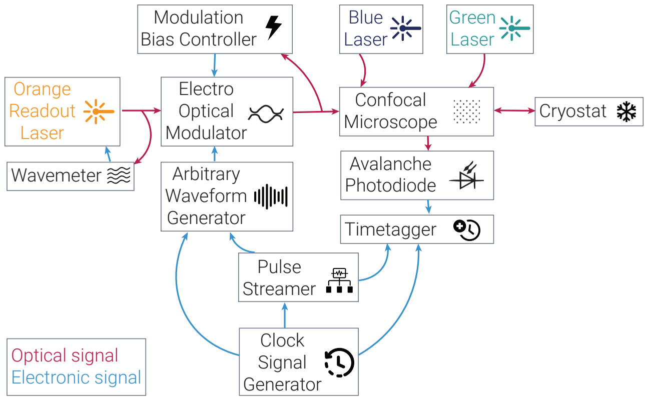

Supplementary Figure 4. **A simplified illustration of the devices and connections that are used to collect signals from SnV electrometers.** See Methods section in the main text for detailed explanations.

RORO data analysis steps

1. Using the periodic calibration signal and a previously measured constant delay between the calibration signal and arrival time of the photons, the starting timestamp with 0 value is determined. The rest of the timestamps are corrected accordingly. Timestamps (ps resolution) are rounded to ns.

2. The 1D timestamp data is organized in a 2D matrix with: The X-axis records 1 to 300 ns (RORO sequence length); the Y-axis indexes the individual RORO sequences; The matrix elements represent: 1 for registered counts, 0 otherwise.

3. We separate the X-axis of the 2D matrix, into three 'frequency windows' according to the elapsed time within a RORO sequence. The first 60 ns is off-resonant ω_0, followed by 120 ns ω_1 (including the ω_{1s} application period), and a final 120 ns ω_2 (including the ω_{2s} application period), following our experimental configuration.

4. Counts in the first 23 ns of each of the five windows are set to 0. This step is required due to the ~10 ns spontaneous emission lifetime of the SnV leaking into the next frequency window.

5. The matrix values are then binned into three data points on the X-axis corresponding to each frequency window.

6. For each registered count, we implement a noise filtering step. The time between consecutive events is compared with previously characterized background count rates. If the inverse time between events is smaller than the background count rate, the earlier event

is rejected and deleted from the matrix. Otherwise, the count is accepted.

7. Consecutive accepted events in the same frequency bin count towards the bright time where the emitter is considered resonant with the corresponding frequency.

8. If there is a period with counts comparable to the background at any frequency window, i.e. a completely dark period when the SnV itself is doubly ionized, is identified, the time period is excluded from the spectral jump analysis.

9. Bright time durations after which the SnV switches to the other resonance for each frequency window are histogrammed. The fit results show negligible changes for bin widths smaller than a characteristic value (found by scanning a range of bin widths). We then choose a bin width smaller than the characteristic value producing the smallest fit uncertainty.

10. The histogram is firstly fit to bi-exponential function. If it fails, the histogram is fit to an exponential function with a single rate.

Selective analysis of bright and dark window rates: In the main text, we compare the jumping rates $\Gamma_{\omega_1 \to \omega_2}$ from a selected resonance during the periods when the registered counts are predominantly received from the investigated resonance ω_1, or the other resonance ω_2. To collect enough statistics for data analysis, we conduct the experiment shown in Fig 3(d) for an extended 90 seconds. Then, we manually select periods when the ω_1 is mainly bright or mainly dark. Next, we combine the bright times in a histogram for both cases. From these histograms, $\Gamma_{\omega_1 \to \omega_2}$ is extracted with a single exponential decay fit.

Note on the extracted fast rates and photon count rate limitations: We attribute the inability to determine the fast rates of several kHz on all of our measurements due to the overall photon count rates of the same order of magnitude. We also note the possibility of a systematic error on the extracted fast rates, as some jumps might not have been registered. This, however, is not a fundamental temporal resolution limitation of our method and is purely related to the photon count rates, as we are already modulating the readout laser at rates faster than a MHz and up to ~ GHz is electronically possible. In Supplementary Table I, we provide demonstrated Purcell enhancement factors and optimized collection rated from the literature, and estimate achievable temporal resolutions while applying RORO sequences.

CONTROL EXPERIMENTS

Verification of emission from a single transition

An important test for our sensor is the verification that the multimodal spectral fingerprint is originating from the same transition. Here, we provide four characterization measurements, under zero magnetic field to exclude Zeeman splitting, that indicate that the signal originates from a single source and transition.

Distribution of jump distances

Among the 19 characterized emitters, hopping distances varying from a few hundred MHz to a few GHz were found. On the investigated samples, either one or two distinct jump processes or their combinations are found, fully consistent with the number of estimated lattice defects. The distribution of these distances is presented in Supplementary Fig. 5a. Therefore, the existence of unknown levels with quasi-forbidden transition rules seems unlikely as the hop distances appear to be random for each emitter.

PL Spectrum

The photoluminescence emission spectrum (Supplementary Fig. 5b measured under 520 nm excitation light at 4 K shows a typical SnV spectrum with discernible spectrometer-limited peaks attributed to C (between levels $|1\rangle$-$|3\rangle$, Fig. 2b from main text) and D ($|2\rangle$-$|3\rangle$) transitions. Since they are ~ 850 GHz apart, we can safely claim that multiple peaks from the PLE scan do not correspond to these transitions.

Autocorrelation measurement

Autocorrelation measurements presented in control experiments for the single-photon ionization charge dynamics model are taken from the emitter investigated in the main text. The likelihood of multiple emitters contributing to the spectrum is made highly improbable by an autocorrelation measurement with $g^{(2)}(0) = 0.12(9) < 0.5$ close to the theoretical expected value of $g^{(2)}(0) = 0$.

Rabi frequencies of different resonances:

Here, we demonstrate Rabi oscillations between levels $|1\rangle$ and $|3\rangle$ (C transition) of an SnV, on emitter E2 at two different resonance frequencies before and after a spectral jump event. In Supplementary Fig. 5c, Rabi frequencies at different powers from both resonances and an example measurement are provided. Oscillations are obtained via resonant excitation after a green stabilization pulse. Data after the resonant laser rise-time is fit to a damped oscillation function. After repeating the measurement at different powers, the lower frequency resonance had a slope of 20.9(9) Hz/$\sqrt{\text{nW}}$, higher frequency one had a 21.2(1.8) Hz/$\sqrt{\text{nW}}$ and combined data set had 21.0(5) Hz/$\sqrt{\text{nW}}$ on a linear frequency-$\sqrt{\text{Power}}$ line. The fact that slopes for three data sets remained within the fitting error range strongly suggests the dipole moment did not

Supplementary Table I. **Measured count rates in the literature, and estimation of achievable RORO acquisition rates. a** Time constants and rates from our experimental configuration. **b** Achievable count rates from literature using nanostructures optimized for enhanced emission and efficient collection. A factor of 2 of the lifetime leaking into the other probing window (see RORO data analysis details), and for 15 & 16 a waveguide to fiber coupling efficiency of 90%[17], detector efficiency of 84.7%[18], quantum efficiency of 80%[19] are assumed for calculating the anticipated acquisition times.

(a)

Specification	Time constant	Rate
SnV lifetime in bulk	5 ns	200 MHz
SnV lifetime in nanopillar	10 ns	100 MHz
RORO acquisition time	60 ns	16.7 MHz

(b)

Reference	Purcell factor	Fluorescence enhancement	Excited state lifetime	Emission rate	Cavity coupling	Setup efficiency	Anticipated acquisition time
20 (NV)	-	-	12.67 ns	79 MHz	-	41%	56.34 ns
15 (SnV)	16	12	0.38 ns	2.63 GHz	95%	-	1.42 ns
16 (SnV)	25	40	0.685 ns	1.46 GHz	90.1%	-	2.62 ns

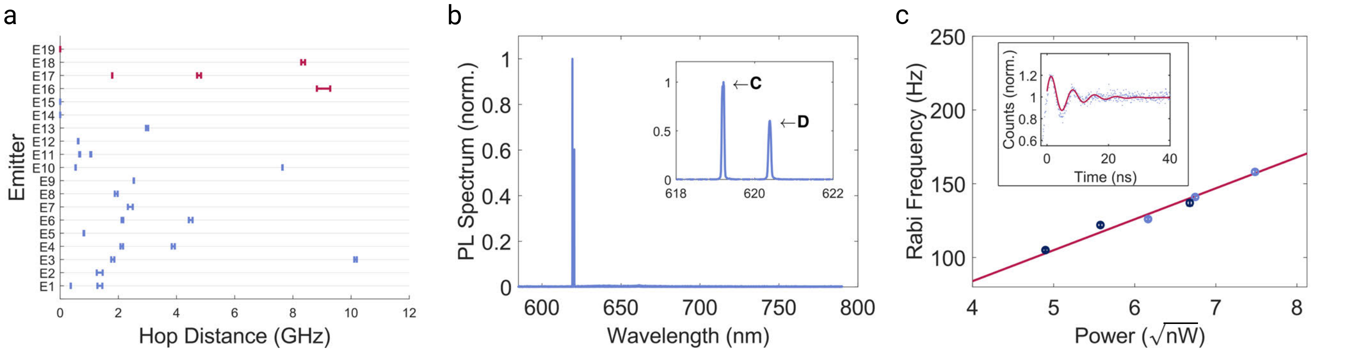

Supplementary Figure 5. **Control experiments to support that the analyzed emission from the SnV belongs to the same transition. a** Spectral hop ranges of characterized emitters. Emitters without hopping were stable during linewidth scans that happened at different time scales between minutes to an hour. Red data points are from a different sample E002 with a higher implantation fluence (2.5×10^{11} cm^{-2}). The error bars show the 95% confidence intervals of the central frequency distances extracted from the fits. **b** Photolimunescence spectrum taken from E1 (emitter from the main text) showing ~850 GHz splitting between C and D transitions. Inset: Zoom-in **c** Rabi frequencies of the emitter E2. Values are extracted from a damped oscillation function at different resonant (between levels $|1\rangle$ and $|3\rangle$) excitation powers. Dark blue and light blue data points are taken before and after a spectral jump, and therefore at different frequencies. Slope of the combined data (red) is extracted as 21.0(5) Hz/ $\sqrt{\text{nW}}$ where the lower frequency (light blue) resonance had a slope of 20.9(9) Hz/ $\sqrt{\text{nW}}$ and higher frequency (dark blue) data is a 21.2(1.8) Hz/ $\sqrt{\text{nW}}$. The uncertainties and error bars represent 95% confidence intervals extracted from the fits. INSET: Example Rabi oscillations observed with 45.5 nW power at the higher frequency resonance.

change between the spectral jumps and the same transition is being addressed between the two measurements.

Demonstration of ionization processes via single-photon processes using autocorrelation measurements

The photophysical picture behind the charge dynamics investigated in the main text has been previously explained in 21. One of the events that can occur during laser irradiation is the group-IV vacancy (G4V) emitters transitioning to a dark state. This manifests as shoulder-like bunching features around the antibunching dip in autocorrelation measurements. Here, applying the single-photon process assumption from the proposed model, we find a linear power dependence for both hole creation/capture and electron promotion processes. These experiments show that the charge transfer picture presented in the main text is consistent with photon statistics measurements. We base our analysis on the derivation of the autocorrelation function and the rate equations provided in the reference 22.

We assume a three level system where level 1 is the ground state, 2 is the excited state, and 3 is a nonradiative shelving state, which is identified as G4V^{-2}. Such a system's $g^{(2)}$ with nonzero background obeys the following equation:

$$g^{(2)} = 1 + p^2[1 - (1 + a)\exp(-\frac{\tau}{\tau_a}) + a\exp(-\frac{\tau}{\tau_b})] \quad (6)$$

where p determines the background contribution, τ_a is the

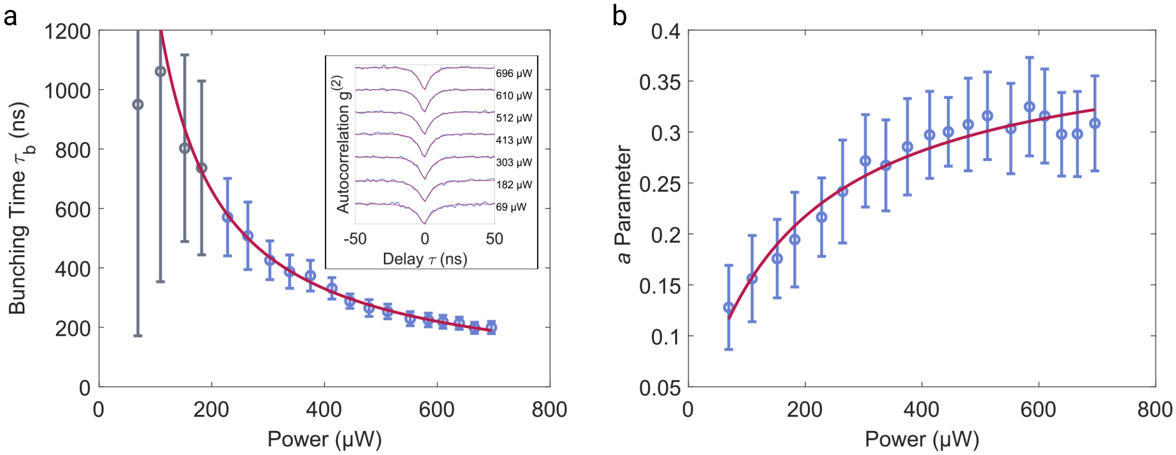

Supplementary Figure 6. **Extracted parameters from the autocorrelation measurements for the emitter E1.** The error bars represent 95% confidence intervals derived from the fits (functions provided in the text). These measurements indicate that shelving and deshelving processes can be modeled as single-photon events. **a** Bunching time at different powers. Grey points are excluded from the fit as they are not expected to behave according to the approximated model at low powers and they have large errors. Solid line: Fit to a $1/(cP)$ function. Inset: Selected example measurements. **b** a parameter at different powers. Solid line: Fit to a saturation curve.

antibunching time related to the dip at 0 delay, τ_b is the bunching time which determines the shoulders surrounding the antibunching dip, and the a parameter is related to the transition rates. To test the model, $g^{(2)}$ measurements of an SnV center at different powers (P) are fit to this equation and the parameters are extracted. The following power relations are then assumed to predict the transition rates ($k_{\mathrm{Initial\,Final}}$):

- k_{12} (incoherent excitation) is assumed to have a linear dependence on power 'δP', as it is a single-photon process promoting an electron from the ground state to quasi-continuous phononic bands of the excited state.

- k_{21} (spontaneous emission) is modelled with a constant rate 'Γ'.

- k_{23} (shelving) is assumed to have a linear power dependence 'αP', because this process is known to be a single-photon process promoting electrons from the valance band to an excited G4V[21].

- k_{31} (deshelving) is also modelled to be linearly proportional to power 'βP': Here, hole donation is assumed to be a single-photon process induced by promoting an electron from valance band to a V_n. Previously, this rate has been modeled with a saturation curve[23], which may be attributed to the limited amount of contributing V_n. However for this case, our Monte Carlo simulations predict too high V_n density for saturation to occur. Therefore, a linear model can capture the data well. We also want to note that a saturation curve imitates a linear relationship at low powers, and both models can work at different regimes consistently.

The bunching time τ_b relates to the transition rates through the equation:

$$\tau_b = \frac{1}{k_{31} + k_{23}\frac{k_{12}}{k_{12}+k_{21}}} \tag{7}$$

If k_{12} is assumed to be much larger than k_{21} – as expected at higher powers –, then $k_{12}/(k_{12} + k_{21})$ approaches 1. Then:

$$\tau_b = \frac{1}{k_{31} + k_{23}} = \frac{1}{(\alpha + \beta)P} \tag{8}$$

This shows that τ_b is effectively determined by the total rate of k_{31} and k_{23} at higher powers. When a 1/x model is fit to the extracted τ_bs at the Supplementary Fig. 6a, it can be seen that the model captures the data well and $\alpha + \beta$ is extracted as 7.5(1) kHz/µW. The total charge cycle rate of 1 MHz at ~ 150 µW also seems reasonable as we expect the charge transfer process to be slower than spontaneous emission or excitation.

For estimating the rate coefficients separately, we can determine the a parameter which is governed by:

$$a = \frac{k_{23}}{k_{31}}\frac{k_{12}}{k_{12}+k_{21}} = \frac{\alpha}{\beta}\frac{\delta P}{\delta P + \Gamma}\,. \tag{9}$$

At high powers, a parameter will asymptotically reach α/β. In Supplementary Fig. 6b, the extracted a values from the measurements follow a saturation curve where the fit asymptotically approaches 0.40(3). Furthermore, using this relation in Eq. (8), we can deduce the shelving and deshelving rates at each power with α = 2.2(2) kHz/µW and β = 5.4(2) kHz/µW. Because the linear power dependence assumption is consistent with the observed data, we argue that single-photon processes are the main driver of the charge dynamics in the sample.

a

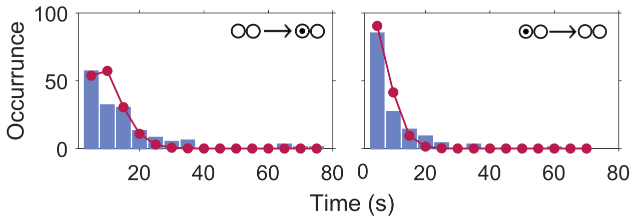

b

$i \to j$	$p(i \to j)$	$\Gamma_{ct}(i \to j)$
$\bigcirc\odot \to \odot\odot$	33(6)%	0.06(1) Hz
$\bigcirc\odot \to \odot\bigcirc$	3(1)%	-
$\bigcirc\odot \to \bigcirc\bigcirc$	45(9)%	0.43(9) Hz
$\bigcirc\odot \to SnV^{-2}$	19(4)%	0.09(2) Hz
$\bigcirc\bigcirc \to \odot\odot$	3(1)%	-
$\bigcirc\bigcirc \to \odot\bigcirc$	23(6)%	0.09(1) Hz
$\bigcirc\bigcirc \to \bigcirc\odot$	59(14)%	0.19(4) Hz
$\bigcirc\bigcirc \to SnV^{-2}$	15(4)%	0.12(2) Hz
$SnV^{-2} \to \odot\odot$	0.9(3)%	-
$SnV^{-2} \to \odot\bigcirc$	0.0(4)%	-
$SnV^{-2} \to \bigcirc\odot$	61(12)%	$\gg\Gamma_s$=0.2 Hz
$SnV^{-2} \to \bigcirc\bigcirc$	38(9)%	$\gg\Gamma_s$=0.2 Hz

Supplementary Figure 7. **Charge dynamics of the sensor S1. a** Example histograms showing the duration resonance remains in a charge state until it switches to another. The data are fit to a Poisson distribution to estimate a mean time. **b** Extracted temporal values from the sensor data presented in Fig. 2b from the main text. $p(i \to j)$ and $\Gamma_{ct}(i \to j)$ represent conditional spectral jump probabilities and rates respectively, where i, j are the initial and final charge state configurations. Missing rates are due to insufficient data points. Γ_s is the scanning rate. The uncertainties are estimated from the overlap of individual peaks for $p(i \to j)$ and 95% confidence intervals extracted from the fits for $\Gamma_{ct}(i \to j)$.

Temporal analysis of the S1 Data

For identifying the position of charge traps, we have used *accumulated* spectral fingerprints that reflect the integrated spectrum for the entire set of charge states $\mathcal{U}_C = \{\bigcirc\bigcirc, \bigcirc\odot, \odot\bigcirc, \odot\odot, \mathrm{SnV}^{-2}\}$, including the dark state SnV^{-2}. Comparing *individual* read-out events of our electrometer, i.e., single PLE linescans between different charge configurations within $\mathcal{U}_C$, gives access to time-resolved charge transfer dynamics.

To characterize the local charge environment and dynamics, we introduce the charge state transition probabilities $p(i \to j)$ and the conditional transfer rates $\Gamma_{ct}(i \to j)$ between charge states i and j of the proximity traps, where $i, j \in \mathcal{U}_C$. We extract $p(i \to j)$ and $\Gamma_{ct}(i \to j)$ by histogramming the charge transfer events and intervals between them (Supplementary Fig. 7a,b). In addition, we define the lifetimes of each configuration as $\tau(i)$.

We begin the analysis by quantifying the smallest $p(i \to j)$. The occurrence of a charge exchange event, considering our present linescan time of 5 seconds, given by $p(\bigcirc\odot \to \odot\bigcirc)$=0.03(1) indicates an improbable direct transfer between the two proximity traps. Moreover, the occurrence of a two-trap charging processes $p(\bigcirc\bigcirc \to \odot\odot)$=0.03(1) is also unlikely, demonstrating that these events are not correlated.

We further explore the relationship between the reinitialization of the bright SnV charge state $\mathrm{SnV}^{-2} \to \mathrm{SnV}^{-1}$[21,24] and the trap's charge states. The probabilities $p(\mathrm{SnV}^{-2} \to \bigcirc\odot)$=0.61(12) and $p(\mathrm{SnV}^{-2} \to \bigcirc\bigcirc)$=0.38(9) are close to the corresponding peak intensities observed in the spectrum (0.63(5) and 0.31(3), respectively), indicating that the trap states are not correlated with the SnV's charge state.

Next, we compare the ionization and neutralization rates for a single trap, $\sum_{X=\bigcirc,\odot} \Gamma_{ct}(\bigcirc X \to \odot X)/2$=0.075(1) Hz and $\sum_{X=\bigcirc,\odot} \Gamma_{ct}(\odot X \to \bigcirc X)/2 \gg \Gamma_s$=0.2 Hz, respectively, with Γ_s the scanning rate. The more than 3-fold higher ionization rate possibly reflects the distinct underlying physical mechanism compared to neutralization. It is notable that the ionization rates of the other trap abruptly change in time: for linescans 0-250 $\Gamma_{ct}(\bigcirc\bigcirc \to \bigcirc\odot)$=0.07(2) Hz and 250 - 500 $\Gamma_{ct}(\bigcirc\bigcirc \to \bigcirc\odot) \gg \Gamma_s$=0.2 Hz. For the neutralization rate, the trend is inverted. We attribute this intriguing change of rates to discrete changes in the trap environment, however, a more detailed analysis is beyond the scope of this work[25]. Furthermore, the distinct ionization rates, $\Gamma_{ct}(\bigcirc\bigcirc \to \bigcirc\odot)$=0.09(1) Hz and $\Gamma_{ct}(\bigcirc\bigcirc \to \odot\bigcirc)$=0.19(4) Hz, observed under the same illumination laser field, indicate either large variations of the local electrostatic potentials in a ~1 nm range modifying charge dynamics or the presence of multiple charge trap species. A future study could therefore help to differentiate among the various V_n.

Lastly, we determine and interpret overall charge state lifetimes $\tau(i)$ which provide a figure of merit for experiments that require spectral stability. We find $\tau(\bigcirc\odot)$ = 2.3(1) s and $\tau(\bigcirc\bigcirc)$ = 4(1) s, approximately corresponding to the duration of a linescan. Our measurement procedure involves a blue 445 nm charge initialization pulse between each linescan, accompanied by continuous orange 619 nm laser illumination. These timescales imply that the primary driver for changes in charge trap states is the blue laser (see Supplementary Fig. 9), suggesting the potential for maintaining trap state stability during optical operations resonant with SnV transitions. Since the trap states are stable much longer than the measured SnV ionization time of 50 ms[21] and spin coherence time of about 1 ms[26], although not deterministically, the emitter may still act as an optically coherent spin-photon interface.

Data analysis steps

1. Wavemeter correction: During scans, the frequency of the laser is controlled by applying an external voltage signal. We monitor the laser frequency through a pick-off path directed at a wavemeter. PLE spectra are initially recorded as a set of voltages and fluorescence signals. The voltage can then be converted to frequencies by matching the time stamps. Any nonlinear frequency changes occurring during the scan are therefore accounted for.

2. Binning: Individual scans are mapped to a frequency axis by selecting an individual linescan and subsequent frequency binning. If multiple data points fall into the same bin, they are averaged. If a bin remains empty, the average of the previous and next bin is used.

3. Histogramming scans: Binned scans are summed and normalized to create the histogrammed PLE spectra.

4. Configuration identification: A peak finder algorithm (MATLAB: findpeaks) is used to identify the frequencies of the four peaks. These peaks are then labeled and used for averaging the spectral location corresponding to a particular charge configuration of proximity traps.

5. Configuration ranges: We separate state configurations by assigning a spectral range to each central peak position. This range is half the spectral distance between two adjacent peaks.

6. Scanwise peak identification: The same peak finder algorithm is used on each individual scan to find peaks.

7. Scanwise configuration identification: The identified peaks are then matched with a charge state configuration depending on their central frequencies.

8. Determining the brighttime durations: The 'brighttime' is determined by the amount of time a peak is associated to the same charge configuration until a change occurs. Each brighttime is recorded together with the changes in the charge state configuration.

9. Histogramming brighttimes: The brighttime durations are histogrammed according to the occurrence they were observed, with the goal of extracting averaged lifetimes and switching rates.

10. Charge state change probability $p(i \to j)$: The amount of times that a spectral jump from a charge state i to another j has occurred is recorded. They are then normalized to the total amount of jumps from configuration i to obtain a probability.

    There are two factors that limit the quantification of uncertainties. First, jumping events depend on the individual identification of peak locations per line. The implemented peak finder algorithm locates the maximum of a line for each scan. Due to spectral diffusion, it is not possible to fit every individual line and extract a central frequency uncertainty. Secondly, there are overlaps of individual spectral peaks in the integrated spectrum in Fig. 2b from the main text. Even though we select a cut-off position in the middle of the peaks, some of the identified peaks could actually belong to the neighboring spectral peak's tail, instead of where we identified its position. Therefore, we assign an overall uncertainty factor by computing the overlap of the individual integrated fits of the individual peaks. We then multiply these factors with the extracted probabilities.

11. Poisson fitting: The histograms are converted to probability densities and then fit with a Poisson distribution. After the fit, the histogram and the fit are scaled back to the original occurrences. The brighttimes are then converted to real time units by the duration of a single scan.

12. Lifetime $\tau(i)$ and conditional spectral jump rate $\Gamma_{\mathrm{ct}}(i \to j)$ extraction: Mean values of the Poisson distribution fits and their uncertainties are provided as proximity charge configuration lifetimes, or their inverse as state switching rates between configurations.

Charge trap-illumination field interactions

In the main text, we show the influence of charge trap densities on spectral diffusion. Here, we provide experimental results on how the properties of the laser can affect spectral diffusion. Since the illumination induces the ionization events in the sample, we show that the interactions and observed phenomena are consistent with the existence of charge traps.

Position dependency of the stabilization laser and subdiffraction drift sensing

A peculiar property of the ZPL of an SnV was reported in 21, Fig. 3B where the spectral line drifted in correlation with the laboratory air conditioning cycle. In order to investigate this further, we conduct simulations to reproduce the periodic changes and inhomogeneous broadening of the reported PLE measurement. Our simulations involve introducing a periodic misalignment of the laser by varying the participating remote charge density.

We assume that the blue stabilization laser has a Gaussian intensity distribution in the z-direction that oscillates in time

$$I(z,t) = I_0 e^{-[z-z_0(t)]^2/2\sigma} , \tag{10}$$

where I_0 is the laser's peak intensity at the focal point, σ the focal width and

$$z_0(t) = a \sin(\omega t) . \tag{11}$$

The amplitude a, describing the magnitude of misalignment due to the changes in temperature of the setup is not known. The frequency $\omega = 2\pi/T$ corresponds to the $T = 10$ min cycle of the air conditioning described in 21. To perform the Monte Carlo simulation we follow the previously outlined steps with the field produced by an ionized trap given by

$$\mathbf{E}(q,\mathbf{r}) = \frac{q_i}{4\pi\epsilon_0\epsilon_r}\frac{\mathbf{r}}{r^3} . \tag{12}$$

We randomly distribute traps with a density of $\rho = 22.7$ ppm in a cubic volume with an edge length of 100 nm. The trap density well reproduces the inhomogeneous broadened linewidth of ≈ 103 MHz shown in Fig. 3b of 21 for a power broadened homogeneous linewidth of ≈ 88 MHz. We assume

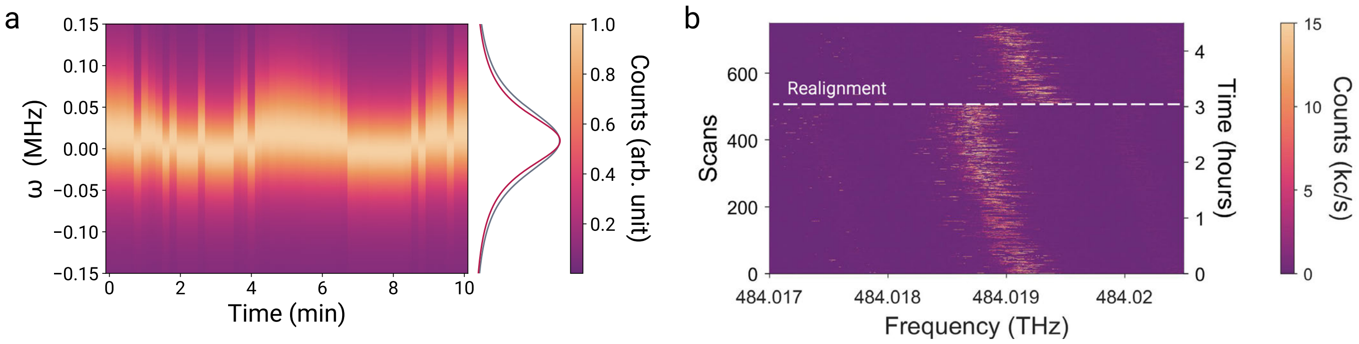

Supplementary Figure 8. **Influence of laser misalignment on the PLE spectra.** **a** Simulation of the temporal change of the central position and linewidth of the C-transition caused by a periodic change in the alignment of the charge state stabilization laser, which can result from temperature changes in the experiment[21]. We assume a power broadened homogeneous linewidth of $\mathrm{FWHM_{hom}}$ = 88 MHz (red line) and find an inhomogeneous linewidth of $\mathrm{FWHM_{hom}}$ = 103.3 MHz (extracted from fit with a Voigt profile, dark gray line) for the charge state polarization laser parameters provided in the main Suppl. text. **b** An exemplary PLE measurement, showing a drift of the resonance frequency. After optimizing the xyz position of the sample and laser spot, the central frequency returns to the original position.

that the probability of a trap participating in being ionized is given by $P(t) = P(z,t) + P_0$, where $P(z,t) \propto I(z,t)$ and $P_0 = 0.1$ is a constant background ionization probability. For the Monte Carlo simulation of the inhomogeneous linewidth at each time step t we use 2500 different charge configurations. We found very good agreement with the results reported in 21 for a laser with a focal spot width of FWHM = 240 nm (σ = FWHM$/2\sqrt{2\log(2)}$) and an oscillation amplitude of a = 200 nm. The results can be seen in Supplementary Fig. 8a.

To further confirm our model, we performed a long-term PLE scan (Supplementary Fig. 8b) and utilized the xyz control of the confocal microscopy setup to optimize the fluorescence signal. By monitoring the changes in the spectral line, we were able to measure a ~200 MHz drift over a 3-hour period, which corresponds to a ~50 nm shift according to our position optimizer. By realigning the setup, we were able to retrieve the initial position of the resonance, providing further evidence in support of our hypothesis.

We extracted spectral drift relations ~0.2 MHz/nm for Supplementary Fig. 8a and ~4 MHz/nm for Supplementary Fig. 8b. This means, depending on the surrounding charge density, it would be reasonable to estimate MHz/nm correspondence of the laser position drift to the emission central frequency. We propose a spectral test such as this could prove useful for estimating the positional drifts under the diffraction limit. It has been demonstrated that chirped pulses from an EOM can scan 200 MHz range under a second[27]. Therefore, using a spectral approach would also allow a higher bandwidth exceeding read-out rates from the fluorescence intensity-based schemes[28].

Overall, an SnV, or emitters with inversion symmetry in general, can be used to temporally resolve the participating remote charge trap density at each moment. By correlating the central frequency, spatial drifts in experimental systems can be tracked.

Stabilization method of the emitter:

We also investigate the spectral properties of our emitters using different charge stabilization procedures involving blue laser light to study its interaction with the V_n. Supplementary Fig. 9 shows PLE scans and spectra using two different stabilization schemes with a charge stabilization laser at 450 nm and 300 nW average power. The first scheme uses continuous-wave (CW) laser light during each PL scan (continuous stabilization). The second is a pulsed scheme: before each PLE scan a blue laser pulse of 4 ms duration irradiates the sample (pulsed stabilization). PLE scans were performed on emitter E1 with a resonant power of 0.7 nW, which lies below the saturation power (> 20 nW), and is also low enough to avoid ionization during the scan.

Supplementary Fig. 9a clearly shows both two resonance peaks (~ 1.4 GHz apart), that are also present in each individual linescan. The individual PLE scans of the pulsed scheme in Supplementary Fig. 9b reveal that both resonances correspond to two distinct spectral positions of the C transition, which we attribute to Stark shift (Eq. (1), main text) induced by two distinct charge configurations of ionized V_n in the vicinity of the SnV. A quasi-continuous fluorescence signal with the resonant laser is observable showing the full inhomogeneous linewidth. Continuous stabilization will cycle the charge state of the environment with a spectral jump rate $\Gamma_{SH} \gg \Gamma_{scan}$ much higher than the PLE scan rate, leading to two recognizable peaks during individual PLE scans.

Another clear signal of the increased ionization of V_n is the more pronounced inhomogeneous broadening of the resonance lines under continuous stabilization. 450 nm CW light will cause more traps in the environment to participate in creating the fluctuating electric field at the emitter's position during each individual scan. Just as predicted by our Monte Carlo simulations, an increased activity of charge traps leads to increased inhomogeneous broadening.

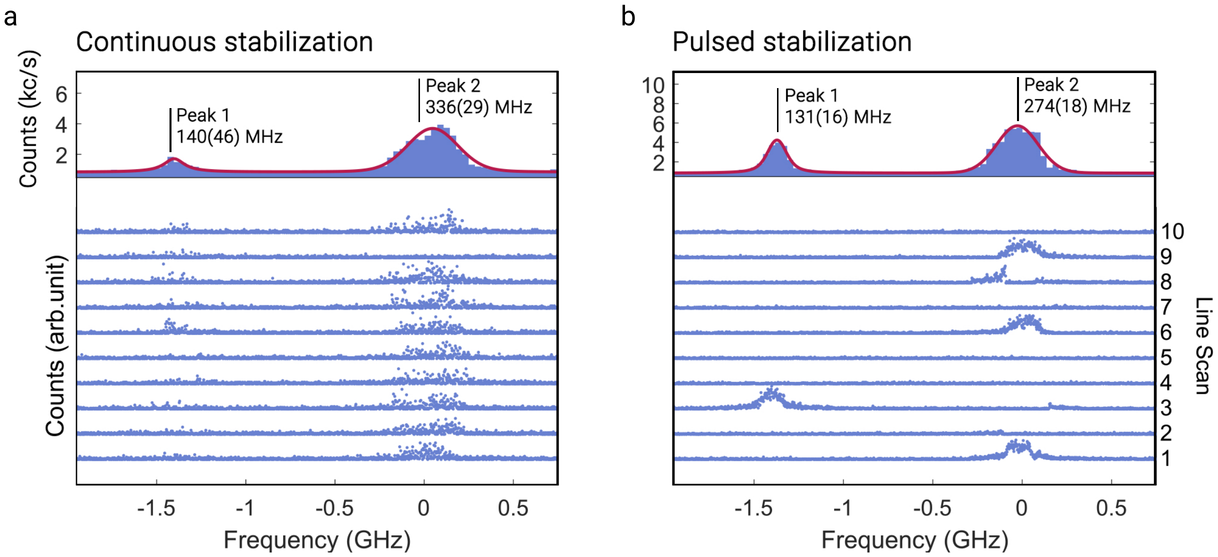

Supplementary Figure 9. **Photoluminescence excitation (PLE) scans of the C transition under different charge stabilization schemes on the emitter E1.** During the scans, a hopping between two different resonances is observed. The resonant laser has a power of 0.7 nW, and the blue laser has a power of 300 nW. The spectra are fit using Voigt profiles. The uncertainties represent 95% confidence intervals extracted from the fits. **a** The blue laser at 450 nm is illuminating the sample continuously during the resonant laser scans. The continuous operation of the blue laser induces hopping that is faster than a single line scan, which results in both peaks being observable in every individual scan. **b** A 4 ms blue laser pulse is sent at the beginning of each scan. Without the help of the blue laser, the cycling between the two resonances is slower but still present due to the laser that is resonant with the C transition.

Wavelength dependency of the stabilization laser:

An indication that charge dynamics and occupation of nearby traps playing a role in the spectral jumping phenomena comes from comparing charge stabilization with blue (450 nm) and green lasers (520 nm). Here in Supplementary Fig. 10, PLE spectra of the emitter E2 taken under the same resonant and stabilization laser powers show a single peaked behavior with the green laser whereas a smaller second peak (although weakly) can be observed when the blue laser is used. It was shown that blue laser irradiation is more efficient for charge trap ionization[21]. Using that information one can deduce that a previously inaccessible charge trap is activated with the blue laser, resulting in the new discrete spectral jump. Spectroscopy of the charge trap transition rates could help in identifying ionization energies for individual charge trap species. For example, it is possible to observe, although only qualitatively, a more quickly switching fluorescence signal within the PLE acquisition resolution with the blue laser at each single line resulting from rapid spectral jumps.

Power dependency of the stabilization laser:

Extended resonant excitation of SnVs induces a dark state transition. This has been connected to a change of its charge state through the promotion of an electron from the valance band. A hole capture process induced by blue or green lasers can reinitialize the SnV back to its bright state. Using higher powers or longer illumination periods increase the probability of charge state stabilization and reemission[21]. This is enabled by ionizing or charging the defects around the quantum emitter which act as charge/hole donors. As a result, illumination changes the charge distribution around the color center and induces spectral diffusion. Therefore, charge stabilization and inhomogeneous broadening become competing effects where one has to optimize both for high quality emission.

In Supplementary Fig. 11, a demonstration of this trade-off in a measurement on emitter E14 is provided. Using a power of 7000 (375) nW results with 30%, 9/30 (23%, 7/30) of the time bright lines and a histogrammed linewidth of 871 (204) MHz. Because charge traps play such a crucial role in the stabilization of the bright state, a competition between spectral diffusion and the charge state stabilization efficiency η_{bright} is expected. The ideal V_n (or hole donors, in general) density can then be determined from a compromise between minimizing spectral diffusion and maximizing η_{bright}.

Additionally, we tried to identify the contribution of blue laser to the spectral diffusion with respect to its power. In Supplementary Fig. 12, we present measurements from the emitter E20, that is on the sample E014 which has a similar fabrication recipe with E001 with five times higher Sn implantation dosage and an extra Sulphur co-implantation step. It can be clearly seen that higher blue laser light powers introduce more pronounced broadening, displaying a degree of saturation, which again is in accordance with both our Monte Carlo simulations and our previous work on spectral diffusion[14].

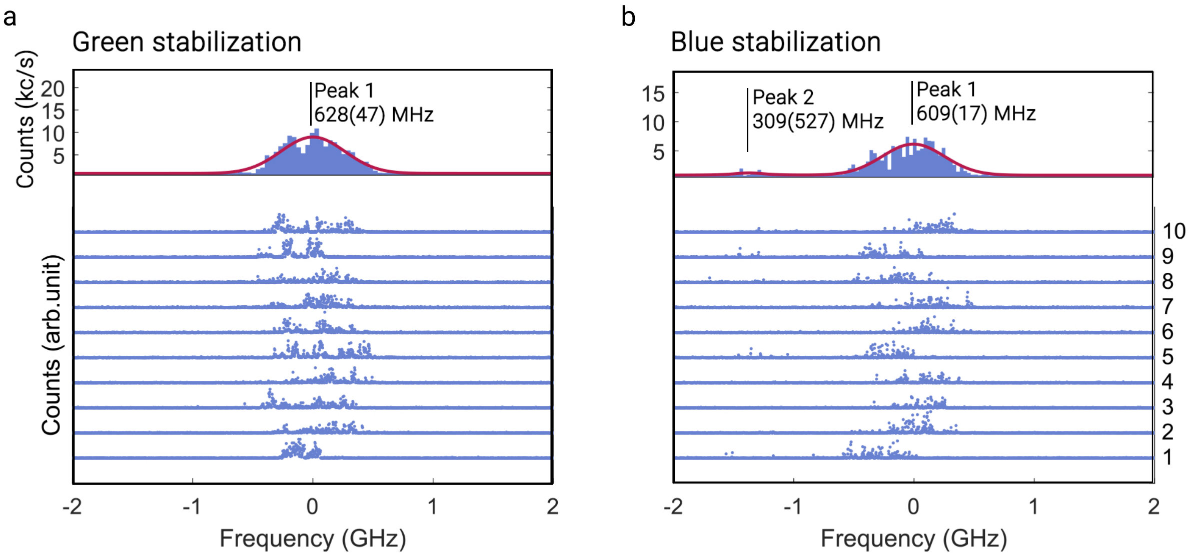

Supplementary Figure 10. **Photolimunescence excitation (PLE) scans of the C transition under different colored stabilization schemes of the emitter E2.** C laser had a power of 1 nW. The spectra were fit using bimodal Voigt profiles. Uncertainties represent 95% confidence intervals extracted from the fits. **a** 500 nW green laser at 520 nm is kept continuously on as the resonant laser scans. Continuous fluorescence from smaller peaks were sometimes observed. The secondary peak was not observable in this configuration. The specta were fit using a Voigt profile. **b** 500 nW blue laser at 450 nm is kept continuously on as the resonant laser scans. The blue laser produces a spectral jump resulting in a secondary peak. The spectrum was fit using a bimodal Voigt profile.

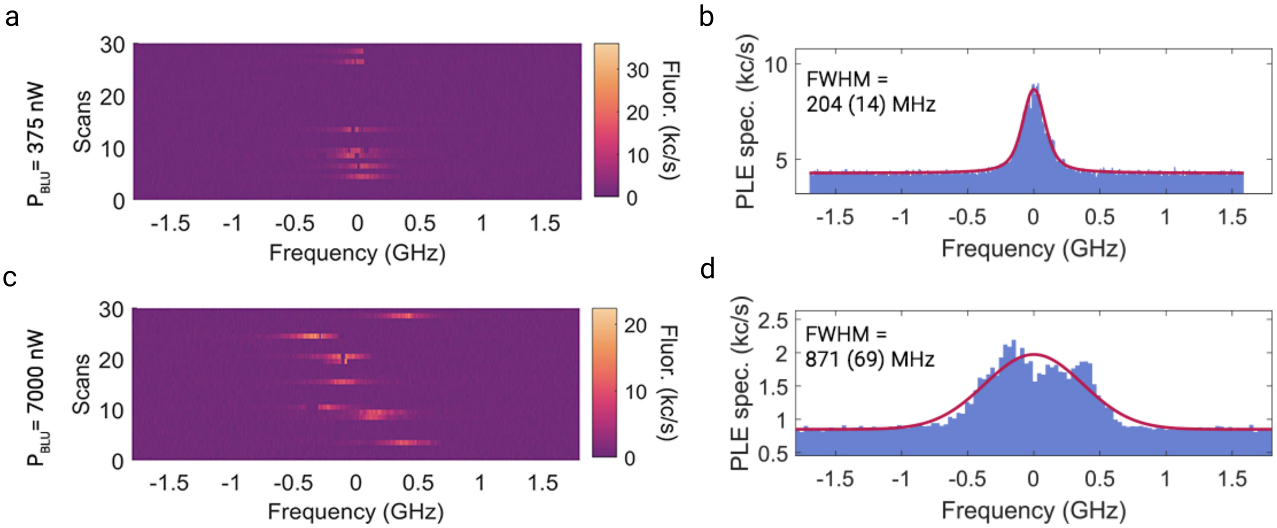

Supplementary Figure 11. **Comparison of different stabilization pulse powers illuminating the emitter E14.** Both measurements are done under 0.5 nW resonant laser excitation. Two different blue laser powers (P_{BLU}) of 375 nW and 7000 nW are used. Higher power resulted in a broadened inhomogeneous linewidth and more pronounced spectral drifts. The spectra were fit using a Voigt profile. The uncertainties represent 95% confidence intervals extracted from the fits. **a**,**c** Measured fluorescence at each cycle as the laser frequency was scanned. **b**,**d** Histogrammed counts for linewidth determination using a Voigt fit.

ADDITIONAL DISCUSSION

Current limitations of the electrometer and how to overcome them

Besides the many exciting opportunities our electrometer offers, we must also critically reflect on the present limitations. In addition to the limited time resolution discussed in the main text, the calibration process can also be improved. We have used an experimentally determined scalar value for the induced polarizability, and not a full tensor, requiring us to infer the direction of the background electric field from consideration of the implantation created defects. The same limitation also prohibits perfectly localizing charge traps in three dimensions.

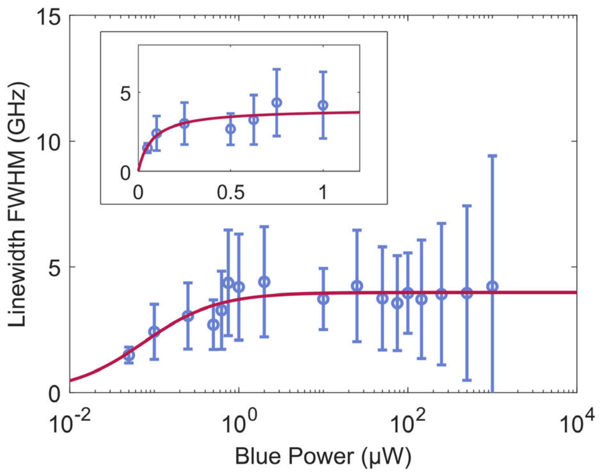

Supplementary Figure 12. **Comparison of different stabilization powers on the emitter E20.** Measurements are taken on another sample which had a five times higher Sn implantation dose compared to the sample used in the main text and was co-implanted with sulfur. The same resonant excitation power of 5 nW is used in all measurements while the power of the blue continuous stabilization laser is varied. Increasing blue power broadens the linewidth reaching an asymptotic limit. The inset shows a zoom-in of the smaller powers to better demonstrate the saturation trend. Error bars are the 95% fit confidence intervals which are heavily influenced by the background fluorescence induced by the comparatively large blue laser powers.

Furthermore, we assume that the position of the observed spectral features is purely caused by induced Stark shifts. We cannot finally exclude any other mechanisms such as strain or phononic contributions, however, the experimental results and our model agree for a variety of local defect configurations, and we therefore conclude that our method is self-affirmative. Any dynamical strain environment cannot also be fully excluded. However, measurements of lifetime-limited lines without any dynamical broadening in implanted samples have been reported[21,29]; therefore, it can be claimed that such an effect did not play a role in previously measured spectra. For the GeV[30], dynamic strain stemming from changes in the Jahn-Teller configuration of surrounding defects has been ruled out as the source of the observed spectra due to a significant mismatch between the predicted and observed hopping rates.

In addition, we have not calibrated the power broadening effects on the natural linewidth of the emission since a saturation measurement is not feasible when a constant signal cannot be retrieved from a spectrally unstable emitter. We can, however, estimate that the effect is negligible since the broadening follows the following equation[31]:

$$\gamma_{\text{broadened}} = \gamma_{\text{natural}} \times \sqrt{1 + \frac{P}{P + P_{\text{saturation}}}} \tag{13}$$

where γ represents the linewidths and P represents excitation powers. Assuming an under-estimated saturation power $P_{\text{saturation}}$ of 10 nW (it was measured to be 4.2 μW in a similarly prepared sample in 32), our measurement power of 0.5 nW corresponds to a broadening less than 2.5%.

Moreover, we are not able to distinguish between all the trap configurations that would yield the same integrated spectral fingerprint. Using the time resolved charge dynamics in conjunction with a detailed physical model of the trap ionization, including the implied charge dynamics and thermalization as well as other possible charge localization mechanisms such as Anderson localization[33] can improve on this ambiguity.

Although the SRIM simulations yield valuable insights into the distribution of V_1 lattice defects during the implantation process, they do not provide an accurate estimation of the post-annealing distribution of higher-order V_n defects. In this study, we take a preliminary step by simulating a V_2 distribution based on the initial V_1 distribution using stochastic methods. However, to obtain more realistic distributions and extend the estimation to multi-vacancy complexes, more advanced techniques such as molecular dynamics simulations, as demonstrated in 34, or incorporating formation energies and diffusion paths on a 3D crystal lattice, as explored in 4, can be employed. These sophisticated approaches have the potential to improve the modeling of realistic distributions and enhance the generalization of estimations for multi-vacancy complexes.

REFERENCES

1. Rayleigh, J. W. S. XXXI. Investigations in optics, with special reference to the spectroscope. *The London, Edinburgh, and Dublin Philosophical Magazine and Journal of Science* **8:49**, 261–274 (1879).
2. Shkarin, A. *et al.* Nanoscopic Charge Fluctuations in a Gallium Phosphide Waveguide Measured by Single Molecules. *Physical Review Letters* **126**, 133602 (2021).
3. Olivero, J. & Longbothum, R. Empirical fits to the voigt line width: A brief review. *Journal of Quantitative Spectroscopy and Radiative Transfer* **17**, 233–236 (1977).
4. Slepetz, B. & Kertesz, M. Divacancies in diamond: a stepwise formation mechanism. *Physical Chemistry Chemical Physics* **16**, 1515–1521 (2014).
5. Fu, X., Xu, Z., He, Z., Hartmaier, A. & Fang, F. Molecular dynamics simulation of silicon ion implantation into diamond and subsequent annealing. *Nuclear Instruments and Methods in Physics Research Section B: Beam Interactions with Materials and Atoms* **450**, 51–55 (2019).
6. Koike, J., Parkin, D. M. & Mitchell, T. E. Displacement threshold energy for type IIa diamond. *Applied Physics Letters* **60**, 1450–1452 (1992).
7. Laidlaw, F. H. J., Beanland, R., Fisher, D. & Diggle, P. L. Point defects and interstitial climb of 90° partial dislocations in brown type IIa natural diamond. *Acta Materialia* **201**, 494–503 (2020).
8. Kiflawi, I., Collins, A. T., Iakoubovskii, K. & Fisher, D. Electron irradiation and the formation of vacancy–interstitial pairs in diamond. *Journal of Physics: Condensed Matter* **19**, 046216 (2007).
9. Newton, M. E., Campbell, B. A., Twitchen, D. J., Baker, J. M. & Anthony, T. R. Recombination-enhanced diffusion of

self-interstitial atoms and vacancy–interstitial recombination in diamond. *Diamond and Related Materials* **11**, 618–622 (2002).

10. Fávaro de Oliveira, F. *et al.* Tailoring spin defects in diamond by lattice charging. *Nature Communications* **8**, 15409 (2017).
11. Ziegler, J. F., Ziegler, M. D. & Biersack, J. P. SRIM – The stopping and range of ions in matter. *Nuclear Instruments and Methods in Physics Research Section B: Beam Interactions with Materials and Atoms* **268**, 1818–1823 (2010).
12. Kambs, B. & Becher, C. Limitations on the indistinguishability of photons from remote solid state sources. *New Journal of Physics* **20**, 115003 (2018).
13. Cui, S. T. Electrostatic potential in cylindrical dielectric media using the image charge method. *Mol. Phys.* **104**, 2993–3001 (2006).
14. Orphal-Kobin, L. *et al.* Optically coherent nitrogen-vacancy defect centers in diamond nanostructures. *Physical Review X* **13**, 011042 (2023).
15. Kuruma, K. *et al.* Coupling of a single tin-vacancy center to a photonic crystal cavity in diamond. *Applied Physics Letters* **118**, 230601 (2021).
16. Rugar, A. E. *et al.* Quantum photonic interface for tin-vacancy centers in diamond. *Physical Review X* **11**, 031021 (2021).
17. Burek, M. J. *et al.* Fiber-coupled diamond quantum nanophotonic interface. *Physical Review Applied* **8**, 024026 (2017).
18. Wang, H. *et al.* Fast and high efficiency superconducting nanowire single-photon detector at 630 nm wavelength. *Applied Optics* **58**, 1868–1872 (2019).
19. Iwasaki, T. *et al.* Tin-vacancy quantum emitters in diamond. *Physical Review Letters* **119**, 253601 (2017).
20. Wan, N. H. *et al.* Efficient extraction of light from a nitrogen-vacancy center in a diamond parabolic reflector. *Nano Letters* **18**, 2787–2793 (2018).
21. Görlitz, J. *et al.* Coherence of a charge stabilised tin-vacancy spin in diamond. *npj Quantum Information* **8**, 1–9 (2022).
22. Kitson, S. C., Jonsson, P., Rarity, J. G. & Tapster, P. R. Intensity fluctuation spectroscopy of small numbers of dye molecules in a microcavity. *Physical Review A* **58**, 620–627 (1998).
23. Neu, E. *et al.* Single photon emission from silicon-vacancy colour centres in chemical vapour deposition nano-diamonds on iridium. *New Journal of Physics* **13**, 025012 (2011).
24. Gardill, A. *et al.* Probing charge dynamics in diamond with an individual color center. *Nano Letters* **21**, 6960–6966 (2021).
25. Bluvstein, D., Zhang, Z. & Jayich, A. C. B. Identifying and mitigating charge instabilities in shallow diamond nitrogen-vacancy centers. *Physical Review Letters* **122**, 076101 (2019).
26. Debroux, R. *et al.* Quantum control of the tin-vacancy spin qubit in diamond. *Physical Review X* **11**, 041041 (2021).
27. Arjona Martínez, J. *et al.* Photonic indistinguishability of the tin-vacancy center in nanostructured diamond. *Physical Review Letters* **129**, 173603 (2022).
28. Mortensen, K. I., Churchman, L. S., Spudich, J. A. & Flyvbjerg, H. Optimized localization analysis for single-molecule tracking and super-resolution microscopy. *Nature Methods* **7**, 377–381 (2010).
29. Narita, Y. *et al.* Multiple tin-vacancy centers in diamond with nearly identical photon frequency and linewidth. *Physical Review Applied* **19**, 024061 (2023).
30. Li, Z. *et al.* Atomic optical antennas in solids. *Nature Photonics* **18**, 1113–1120 (2024).
31. Cohen-Tannoudji, C., Dupont-Roc, J. & Grynberg, G. *Atom—Photon Interactions* (Wiley, 1998).
32. Trusheim, M. E. *et al.* Transform-limited photons from a coherent tin-vacancy spin in diamond. *Physical Review Letters* **124**, 023602 (2020).
33. Anderson, P. W. Absence of Diffusion in Certain Random Lattices. *Physical Review* **109**, 1492–1505 (1958).
34. Lehtinen, O. *et al.* Molecular dynamics simulations of shallow nitrogen and silicon implantation into diamond. *Physical Review B* **93**, 035202 (2016).
